# Discharge AI-ECG-Derived Age and Incidence of Cardiac Allograft Vasculopathy at 3 Years: A Pilot Study

**DOI:** 10.3390/jcdd13080400

**Published:** 2026-08-20

**Authors:** Andrea Muniz, Yugant Khand, Vidit Yadav, Aarti Desai, Laxmi Raj Bangari, Surbhi Dadwal, Jose Ruiz, Devora Leventhal, Rohan Goswami

**Affiliations:** Department of Heart Failure and Transplant, Mayo Clinic Florida, Jacksonville, FL 32224, USA; muniz.andrea@mayo.edu (A.M.); dr.yugantkhand@gmail.com (Y.K.); yadav.vidit@mayo.edu (V.Y.); desai.aarti@mayo.edu (A.D.); bangari.laxmiraj@mayo.edu (L.R.B.); dadwal.surbhi@mayo.edu (S.D.); ruizmorales.jose@mayo.edu (J.R.); leventhal.devora@mayo.edu (D.L.)

**Keywords:** artificial intelligence, electrocardiogram, biological age, risk predictor, cardiac allograft vasculopathy

## Abstract

Cardiac allograft vasculopathy (CAV) remains a leading cause of graft failure after heart transplantation, yet an early, non-invasive risk stratification tool is not available. Artificial intelligence electrocardiography (AI-ECG) can estimate biological heart age, and it has been associated with cardiovascular risk. Its deviation with chronological age, called delta age, and its utility in the prediction of CAV has never been explored. We conducted a retrospective single-center study for heart transplant recipients bridged with Impella 5.5 (Abiomed, Danvers, MA, USA) at Mayo Clinic Florida (2020–2023). ΔAge was calculated at discharge and 1-year post-transplant. The primary outcome was confirmed CAV at 1 year. Univariate and multivariable logistic regression models were used to assess the independent association between the ΔAge and the development of CAV, with discrimination evaluated by AUC and internally validated via bootstrap resampling (2000 iterations). Fifty patients were reviewed. CAV developed in 13 (26%) patients in 3 years. CAV+ patients had a significantly higher ΔAge at discharge compared to CAV- patients (median −1.0 vs. −11.0 years, *p* = 0.0023) and at 1 year (median +7.0 vs. −4.0 years, *p* = 0.0080). On univariate analysis, ΔAge at discharge was a significant predictor of the development of CAV (OR 1.084 per year, AUC 0.788). On multivariable adjustment, ΔAge at discharge remained an independent predictor (adjusted OR 1.138, 95% CI 1.023–1.265, *p* = 0.018, and model AUC 0.859). ΔAge at discharge is a novel and potential predictor of the development of CAV within 3 years. These findings support the integration of AI-ECG into post-transplant surveillance as a scalable, low-cost tool.

## 1. Introduction

Cardiac allograft vasculopathy (CAV) continues to be a leading cause of late graft failure and mortality after heart transplantation. It affects 30% of recipients at 5 years and 50% at 10 years [1]. Current diagnostic strategies, such as invasive coronary angiography, optical coherence tomography or intravascular imaging, have suboptimal sensitivity for early-stage detection, along with the added procedural risk and increased cost [2].

Non-invasive imaging has become central to CAV surveillance. CAV affects both epicardial vessels and microvasculature in a diffuse and concentric manner, which is better visualized with quantitative flow techniques (positron emission tomography, cardiac magnetic resonance imaging) and high-resolution anatomic imaging (coronary computed tomography angiography) [3,4]. Non-invasive imaging has proven to help the interval between invasive surveillance studies. Coronary CT angiography offers high sensitivity (~97%) but limited serial contrast-related renal risk [5]. PET and MRI have shown strong diagnostic and prognostic accuracy for CAV, including high negative predictive value for excluding significant disease, though validation data are largely from later post-transplant years and neither yet replaces invasive angiography for early detection [6].

Over the past decade, there have been necessity-driven advancements in transplant care, focusing on improving donor–recipient matching, lowering rejection, optimizing lifelong immunosuppressive regimens, and developing novel techniques for less-invasive surveillance [7]. New non-invasive physiological screening options, including myocardial blood flow reserve and donor-derived cell-free DNA for rejection surveillance, have emerged; unfortunately, their prognostic value remains limited [2,8,9].

A scalable, low-cost biomarker for early CAV risk stratification remains an unmet need. Recently, biological age, rather than chronological age, has gained attention as a predictive tool for morbidity and mortality across specialties. Biological age provides a health snapshot and is influenced by epigenetic, cellular, genomic, and environmental factors. With advances in artificial intelligence (AI) and deep learning, electrocardiograms (ECGs) can now predict biological age [10]. AI electrocardiography (AI-ECG) has demonstrated the capacity to extract biological signals from standard 12-lead ECG data, enabling detection of structural heart disease, prediction of mortality, and cardiac biological age estimation [7,11]. The original validated AI-ECG age algorithm achieved a mean absolute error of 6.9 ± 5.6 years against chronological age (R^2^ = 0.7) [12]. Research has focused on the deviation between chronological age and AI-ECG age, known as delta age (ΔAge). A recent meta-analysis of 17 studies found that elevated ΔAge was associated with significantly increased risk of all-cause mortality (HR 1.62, 95% CI 1.49–1.77) and cardiovascular mortality (HR 2.12, 95% CI 1.71–2.63) [10,11]. Despite this, the application of AI-ECG-derived ΔAge in the post-transplant population and specifically in predicting CAV has not yet been explored.

In this pilot study, we investigated whether AI-ECG-derived Δ*Age* at relevant post-transplant timepoints is linked with CAV development at 3 years in a cohort of heart transplant recipients with Impella 5.5 (Abiomed, Danvers, MA, USA) support. We hypothesized that an increased Δ*Age* reflecting an older allograft relative to recipient age would be independently associated with higher CAV risk and provide incremental prognostic value beyond conventional clinical comorbidities. Notably, AI-ECG represents a non-invasive, inexpensive and easily accessible modality, making it a promising surveillance tool in this high-risk population.

## 2. Materials and Methods

### 2.1. Study Design and Population

A single-center retrospective cohort study of fifty consecutive adult heart transplant recipients between January 2020 and 2023 captured all patients with three-year follow up who had a 12-lead electrocardiograph (ECG) at discharge. Among 163 patients who underwent heart transplantation following Impella 5.5 support at our center between 2020 and 2025, 113 were transplanted after 2023 and therefore did not meet the predefined study window for 3-year follow-up. The remaining 50 patients were eligible for inclusion, and all 50 had a 12-lead ECG available at hospital discharge. The study was conducted in line with the STROBE guidelines.

### 2.2. AI-ECG Age Assessment

The biological heart age was estimated using a validated deep-learning AI-ECG algorithm [7], which inputs standard 12-lead ECG waveform data and outputs an age estimate, which we call the AI-ECG Age. The algorithm is a convolutional neural network that processes the raw digital waveform directly, using stacked convolutional and pooling layers to extract temporal and spatial patterns across all 12 leads, which are then fused and passed through a fully connected output layer to generate a continuous age estimate. The pretrained algorithm was applied to the discharge ECGs in the present cohort without retraining, recalibration, or modification using study-specific data [6]. We then utilized AI-ECG Age to define the heart age gap (ΔAge) using the following equation:

Δ*Age* at discharge = *Discharge AI-ECG age* (y) − *Recipient chronological*

*age* (y) at discharge

A positive ΔAge value indicated that the transplanted heart demonstrated a biological older age than chronological age, whereas negative values indicate a biologically younger allograft. The serial AI-ECG measurements were available in 47 patients (94%) at 1 year and in 38 patients (76%) at 3 years, resulting from loss-to-follow-up and censoring.

### 2.3. Outcomes

The primary endpoint was a 3-year time-to-first composite endpoint of (1) angiographically confirmed cardiac allograft vasculopathy or (2) all-cause mortality. Cardiac allograft vasculopathy was assessed by our institution protocol for surveillance with coronary angiography performed at 1, 2, and 3 years post-transplant. CAV severity was graded according to International Society for Heart and Lung Transplantation (ISHLT) criteria, which classify disease based on angiographic findings into ISHLT grade 0 (no detectable disease) through grade 3 (severe), incorporating left ventricular systolic function and the presence of diffuse or focal stenoses ≥70% in the left main or two or more epicardial vessels. In this study, any angiographically confirmed CAV (ISHLT grade 1–3) was considered a positive event for the primary composite and CAV-specific secondary endpoint [13]. All patients at our institution receive coronary angiography as standard of care within the first 5 years post transplantation for CAV surveillance. Patient-specific assessment for invasive testing is performed after 5 years.

Time zero was defined as the date of hospital discharge following hospitalization during the transplant episode of care.

Secondary endpoints were cardiac allograft vasculopathy alone and all-cause mortality alone. All events were adjudicated by three independent reviewers to screen the electronic medical report for catheterization and mortality reports. The earliest event time was used for composite endpoints in case both cardiac allograft vasculopathy and morality occurred.

### 2.4. Statistical Analysis

We reported continuous variables as median (interquartile range) for non-normally distributed data (assessed by Shapiro–Wilk test) and mean (standard deviation) for normally distributed data. The categorical variables are presented as counts and percentages. We used the Mann–Whitney U test, Fisher exact test, or chi-square test as appropriate for between-group comparisons. We reported the standardized mean differences (SMDs) to quantify the imbalance in each group.

We analyzed the time-to-event using the Cox proportional hazard model. The primary analysis evaluated the univariate association between ΔAge and composite endpoints. We maintained an adjusted model using ΔAge, recipient chronological age, and donor age with rigid penalization (λ = 0.10) to avoid overfitting due to limited number of events (*n* = 16). The proportional hazard assumption for Cox regression models was evaluated graphically using Schoenfeld residuals. We also tested whether the relationship between ΔAge and the outcome was non-linear using restricted cubic spline logistic regression. The reverse Kaplan–Meier method was stratified by median ΔAge (−7 years) but is only labeled exploratory in our analysis.

The cumulative incidence was estimated using the Aalen–Johansen estimator for CAV-specific analysis with bootstrap resampling (1000 resamples) for internal validation of model discrimination. A time-dependent receiver operating characteristics analysis with the area under the curve was calculated using 95% confidence intervals by bootstrap. We used the Youden index to identify the ΔAge cutoff for predicting outcomes. The analyses were conducted in Python 3.12. All tests were two-sided, and a threshold of α = 0.05 was noted. We emphasized the effect-size estimation and confidence interval over *p*-value given the exploratory nature of the study.

## 3. Results

### 3.1. Patient Characteristics

A total of 50 adult patients (mean recipient age 59.1 ± 12.5 years; 84% male) who underwent heart transplantation following Impella 5.5 bridging between January 2020 and 2023 were included in the study. The baseline characteristics are presented in Table 1.

The cohort was predominantly male and white, with common comorbidities including hypertension (66%), diabetes (46%), and ischemic cardiomyopathy (38%). Over the 3-year observation period, 16 patients (32%) experienced the primary composite endpoint of which 12 (24%) developed CAV of ISHLT Grade 1 or higher, and four (8%) died.

### 3.2. Distribution of Discharge AI-ECG ΔAge

**Figure 1:**
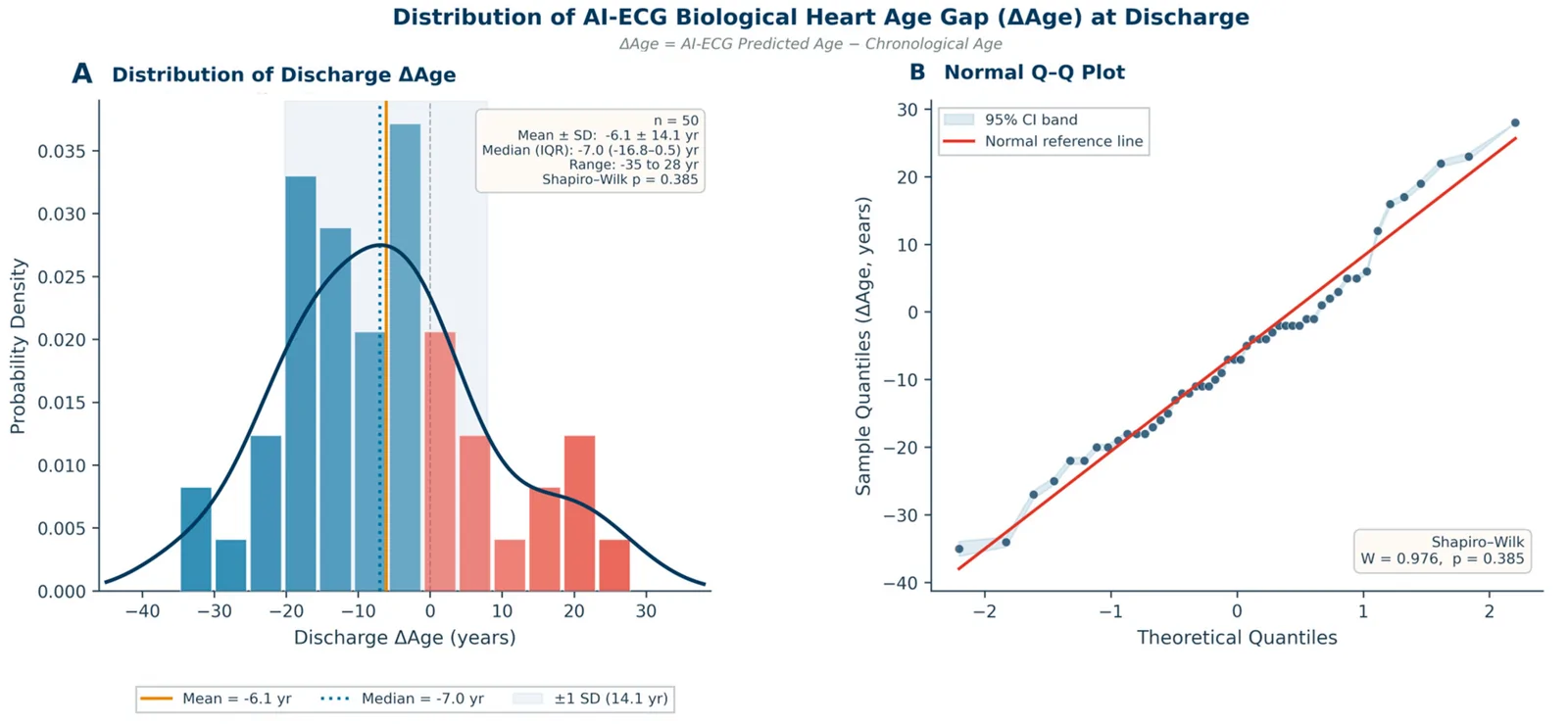
Distribution of discharge AI-ECG biological heart age gap (ΔAge). ΔAge was calculated as AI-ECG predicted age minus recipient chronological age at the time of discharge. (**A**) Histogram showing the distribution of discharge ΔAge across the cohort (*n* = 50). Blue bars represent negative ΔAge values (biologically younger allograft relative to recipient age); red bars represent positive ΔAge values (biologically older allograft). The solid orange line denotes the mean (−6.1 years); the dotted line denotes the median (−7.0 years, IQR −16.8 to 0.5); the shaded band denotes ±1 SD (14.1 years). The distribution did not significantly deviate from normality (Shapiro–Wilk *p* = 0.385). (**B**) Normal quantile-quantile (Q-Q) plot of discharge ΔAge, with the red line representing the theoretical normal reference and the shaded band representing the 95% confidence interval, further supporting approximate normality of the ΔAge distribution (W = 0.976, *p* = 0.385).

The discharge ΔAge ranged from −35 to +28 years across the cohort (mean: −6.1 ± 14.1 years; median: −7.0 years, Figure 1A). The distribution was approximately normal (Shapiro–Wilk *p* = 0.385; Figure 1B). Patients who subsequently developed the composite endpoint had an increased mean discharge ΔAge compared with event-free patients (−1.8 ± 14.0 vs. −8.0 ± 13.9 years; SMD 0.45; *p* = 0.091).

### 3.3. Primary Analysis: Composite Endpoint

In univariable Cox proportional hazard regression, each SD increase (14.2-years) in discharge ΔAge was associated with an 81% higher hazard of the composite endpoint (HR 1.81, 95% CI 1.16–2.83; *p* = 0.009; Table 2).

**Figure 2:**
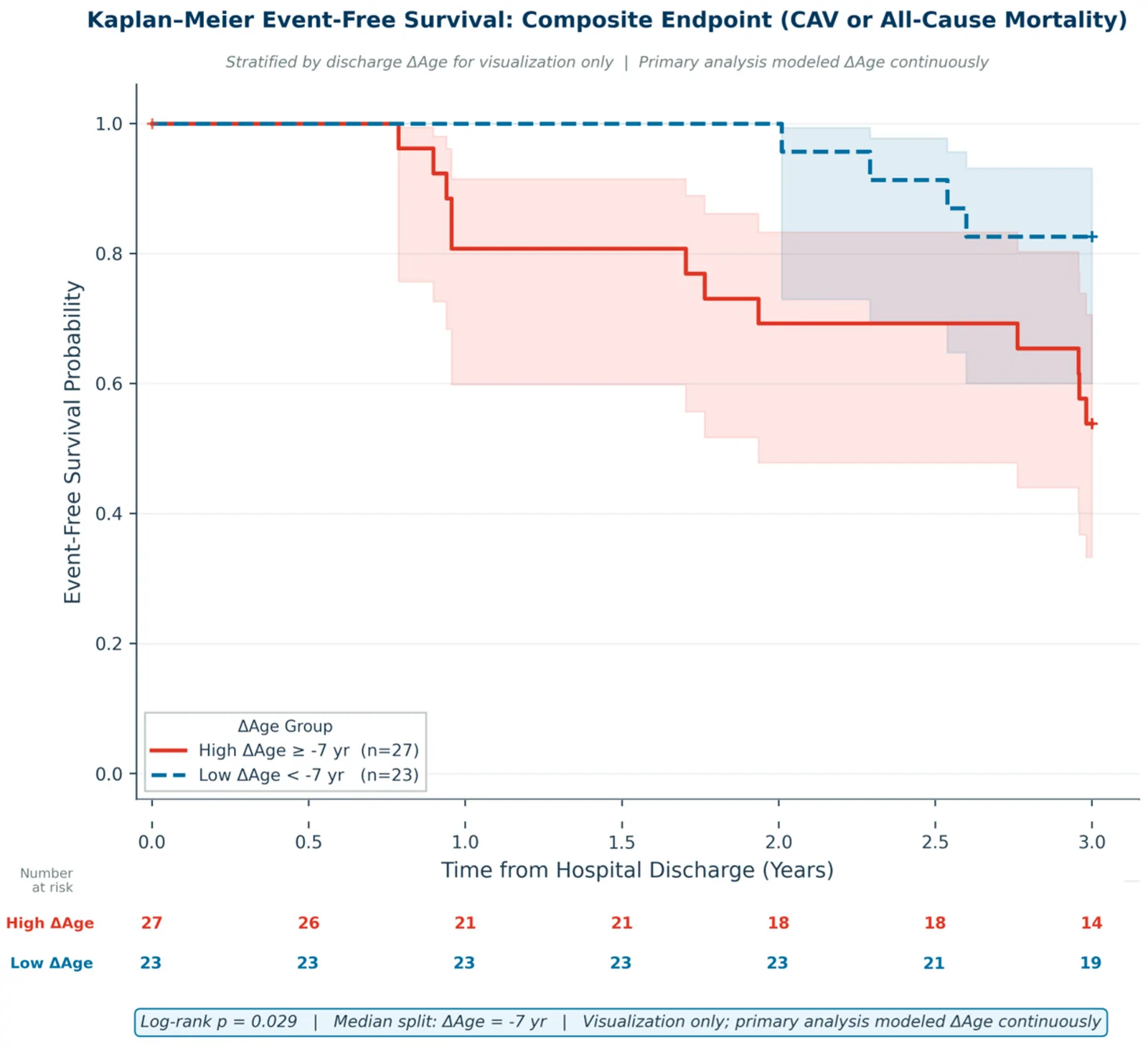
Kaplan–Meier survival curves stratified by discharge ΔAge. The solid red line represents patients with high ΔAge (≥−7 years, *n* = 27); the dashed blue line represents patients with low ΔAge (<−7 years, *n* = 23). Shaded bands denote 95% confidence intervals. Patients with high discharge ΔAge demonstrated lower event-free survival across the 3-year follow-up period (log-rank *p* = 0.029). Numbers at risk at each time point are shown below the x-axis.

Kaplan–Meier survival curves stratified by median ΔAge demonstrated a higher cumulative incidence of the composite endpoint in the higher ΔAge group across the 3-year follow-up period (log-rank *p* = 0.029; Figure 2). The proportional hazard assumption was not violated according to the global scaled Schoenfeld residual test (*p* = 0.65), supporting the appropriateness of the Cox regression framework for this analysis.

### 3.4. Multivariate Analysis

The association between discharge ΔAge and the composite endpoint was attenuated but directionally consistent (HR 1.47, 95% CI 0.87–2.47; *p* = 0.147; Table 2) in an adjusted multivariate model. These findings should be interpreted with appropriate caution as exploratory and hypothesis-generating due to limited events-per-variable (16 events, three covariates).

### 3.5. Secondary Analysis: CAV-Specific Endpoint

**Figure 3:**
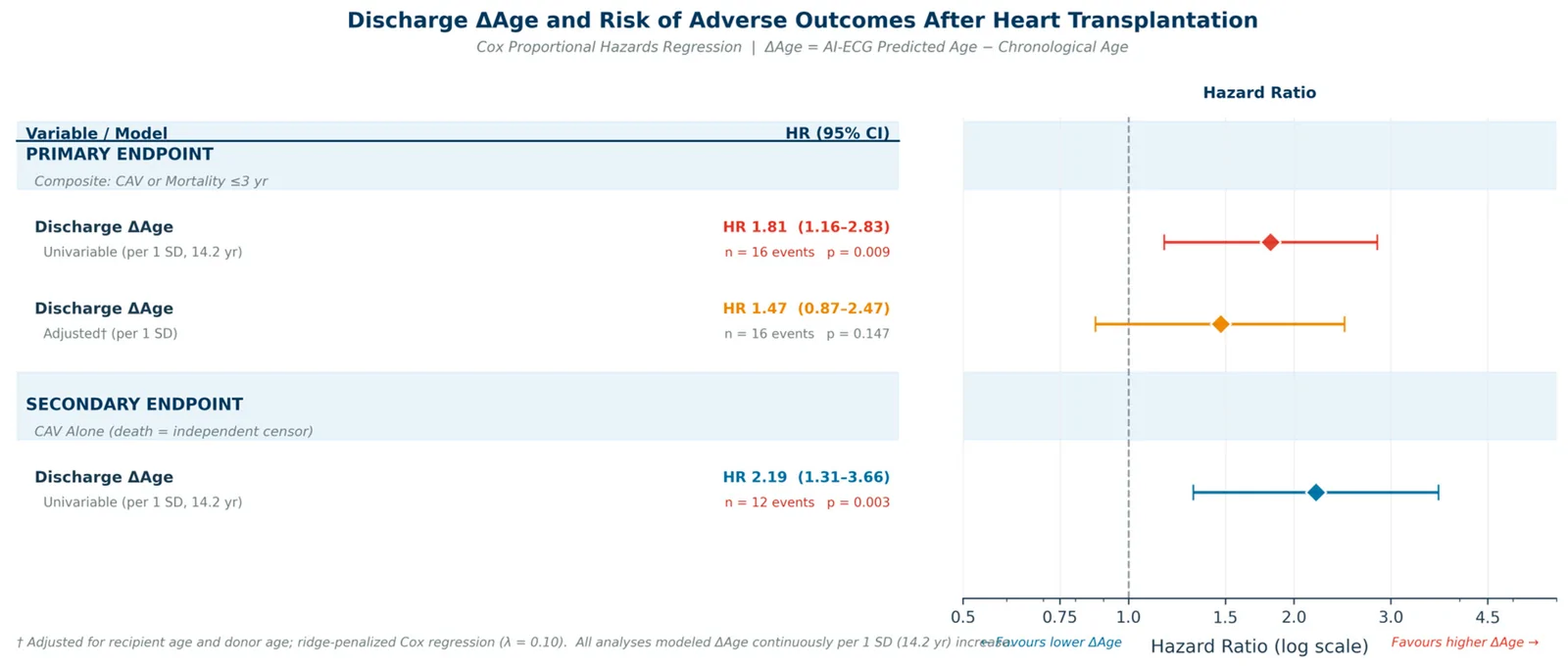
Forest plot of hazard ratios for discharge ΔAge and risk of adverse outcomes after heart transplantation. Hazard ratios (HRs) are reported per 1-SD increase (14.2 years) in discharge ΔAge. For the primary composite endpoint (CAV or all-cause mortality, *n* = 16 events), the univariable HR was 1.81 (95% CI 1.16–2.83, *p* = 0.009; red diamond), attenuating to 1.47 (95% CI 0.87–2.47, *p* = 0.147; orange diamond) after adjustment for recipient age and donor age using ridge-penalized Cox regression (λ = 0.10). For the secondary CAV-alone endpoint (death treated as an independent censor, *n* = 12 events), the univariable HR was 2.19 (95% CI 1.31–3.66, *p* = 0.003; blue diamond). The dashed vertical reference line denotes HR = 1.0. Error bars represent 95% confidence intervals.

The univariate association with discharge ΔAge was stronger than for the composite endpoint (HR 2.19, 95% CI 1.31–3.66; *p* = 0.003; Table 2, Figure 3) when restricted to CAV-specific endpoint (*n* = 12 events; death treated as an independent censor). This finding was directionally consistent across all sensitivity analyses (Table 3).

### 3.6. Discrimination, Calibration, and Internal Validation

The apparent Harrell C-index for discharge ΔAge predicting the composite endpoint was 0.692. The estimated optimism was negligible with an optimism-corrected C-index of 0.692 after internal validation of 1000 resamples (Table 3).

**Figure 4:**
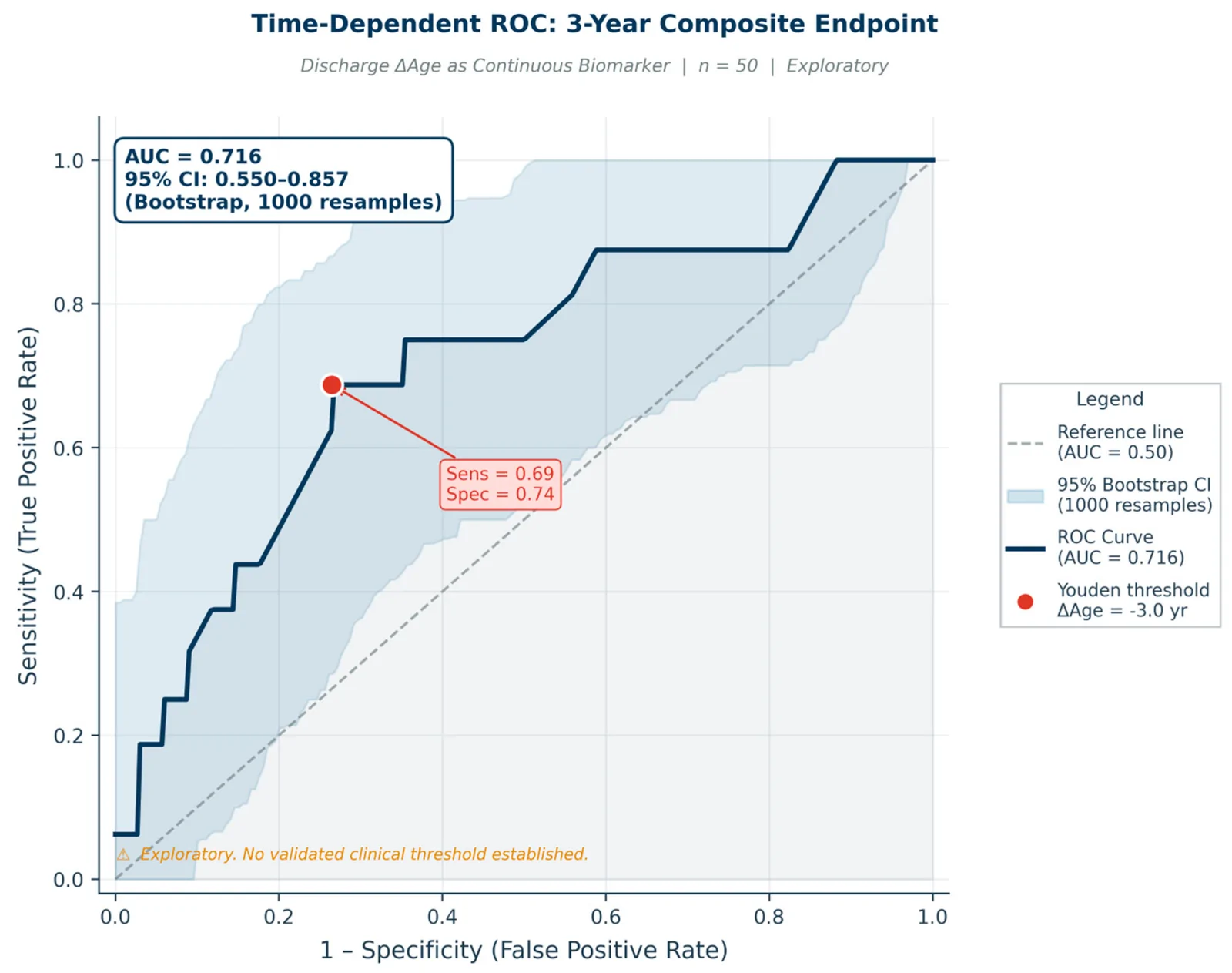
Time-dependent receiver operating characteristic curve for 3-year composite endpoint. The solid navy line represents the ROC curve (AUC = 0.716, 95% CI 0.550–0.857, derived from 1000 bootstrap resamples); the shaded band represents the bootstrap 95% confidence interval. The diagonal dashed line represents the line of no discrimination (AUC = 0.50). The red point marks the Youden-index optimal threshold (ΔAge = −3.0 years; sensitivity 0.69, specificity 0.74).

**Figure 5:**
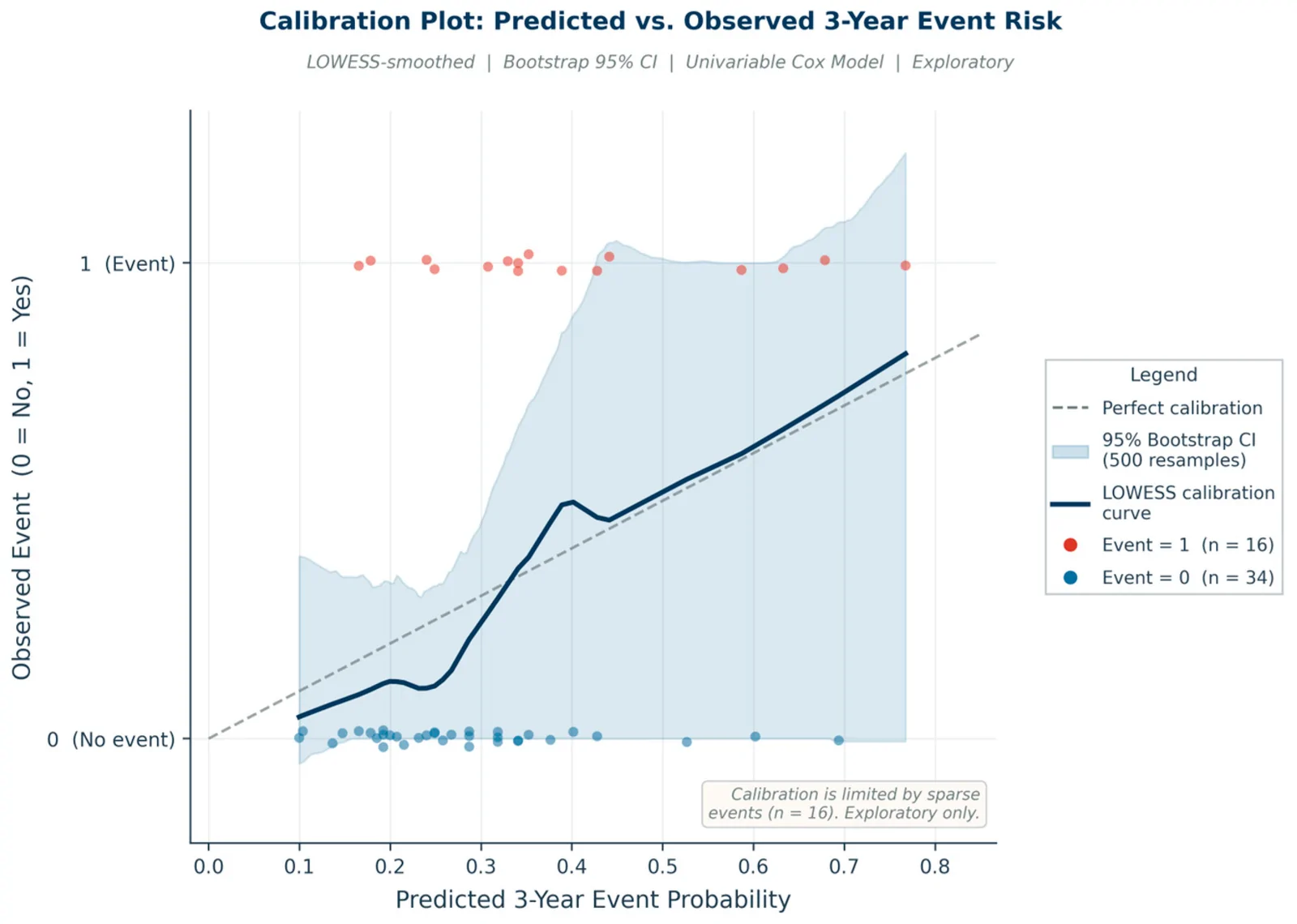
Bootstrap-smoothed calibration plot for predicted vs. observed 3-year event risk. Predicted 3-year event probabilities (x-axis) are plotted against observed outcomes (y-axis; 0 = no event, 1 = event). Red points represent patients who experienced the composite endpoint (*n* = 16); blue points represent event-free patients (*n* = 34). The solid navy line represents the LOWESS-smoothed calibration curve, with the shaded band denoting the bootstrap 95% confidence interval (500 resamples). The dashed diagonal line represents perfect calibration.

The time-dependent ROC analysis at 3 years yielded an AUC of 0.716 (95% CI 0.550–0.857) showing moderate discriminative performance in this exploratory pilot cohort (Figure 4). An exploratory Youden-index optimal threshold of ΔAge = −3.0 years was identified but is strictly hypothesis-generating and should not be applied clinically or used for decision-making in the absence of prospective validation. The calibration is limited by sparse events (*n* = 16). These results are presented in Figure 5 with appropriate caveats. The directional association between higher discharge ΔAge and greater adverse event risk was consistent across all pre-specified sensitivity analyses (Table 3).

## 4. Discussion

In this pilot study of heart transplant recipients, bridged with Impella 5.5 support, an increased AI-ECG-derived biological age gap at discharge was independently associated with an increased risk of cardiac allograft vasculopathy at 3 years. This coronary artery disease, specific to heart transplant recipients, represents a major challenge in this population. Despite decades of research into its complex pathophysiology, CAV remains without a validated non-invasive tool for early detection. The application of AI-ECG-derived Δ*Age* represents a new direction in transplant cardiology. While it has been previously studied as a non-invasive tool for predicting allograft rejection and cardiovascular risk in the general population, its utility in predicting long-term graft vasculopathy has never been reported [14].

Recent evidence has highlighted the complexity of CAV, whose pathogenesis remains with significant knowledge gaps. Its progression is shaped by distinct trajectories and molecular drivers [15]. Loupy et al. performed a large prospective study identifying key clinical predictors, including donor age, acute cellular rejection, and recipient dyslipidemia to define a trajectory-based CAV risk stratification in heart transplant recipients [8]. However, these trajectories remain dependent on invasive angiographic surveillance.

Discharge was selected as our main timepoint as it represents a physiologically stable state, following resolution of cardiogenic shock, discontinuation of any temporary mechanical support devices, and weaning of inotropic therapy, all of which could influence the AIECG parameters. At this timepoint, the transplanted heart has begun to adapt to its new environment. The fundamental question driving this analysis was whether the donor’s heart, once transplanted into a recipient of different chronological age, adapts its electrocardiographic age to match its new host. More importantly, early deviation from that adaptation signals future allograft injury.

Higher Δ*Age* has been previously associated with a range of findings including abnormal ECG patterns such as atrial fibrillation, left bundle branch block, atrioventricular block and prolonged QT interval as well as an unhealthy lifestyle including physical activity, smoking and alcohol consumption. Unsurprisingly, established pathologies such as ischemic heart disease and heart failure have been linked to increased heart age [11]. Given this landscape, the role of this non-invasive, not painful and readily accessible marker in heart transplant patients warranted exploration. Our findings offer the alternative to using a standard 12-lead ECG as an initial approach for risk stratification.

Across our cohort, AI-ECG Δ*Age* at discharge was independently associated with 3-year CAV development, with the strongest signal observed in the CAV-specific analysis (HR 2.19, *p* = 0.003), which suggests that age may be particularly relevant to CAV injury rather than mortality. However, substantial overlap and nonsignificant between-group differences indicate limited discriminative value at the individual level. The AUC of 0.716 suggests moderate discrimination. Thus, ΔAge should be viewed as a population-level prognostic marker rather than a stand-alone diagnostic tool. Given the low event rates of CAV in this single center study, we hope that prospective validation may complement established clinical and imaging risk factors. We identified the Youden threshold of 3 years but require prospective validation. An increased Δ*Age* at discharge may reflect subclinical graft dysfunction or donor-related injury not captured by conventional parameters. The AI-ECG-derived age represents an ECG-based age estimate of the recipient and should not be interpreted as a direct measurement of donor cardiac age.

The association between increased Δ*Age* and CAV risk suggests that AI-ECG has a clinical correlation of graft health in the immediate post-transplant period. The negative mean discharge Δ*Age* across the cohort (mean −6.1 years) indicates that the transplanted allograft appeared biologically younger than the recipient’s chronological age at discharge, consistent with the expected age difference between donors and recipients. The AI-ECG-derived age gap is likely capturing a biological signal that extends beyond chronological age alone, reflecting the cumulative effects of hemodynamic stress, immune-mediated injury, graft-recipient interaction, subclinical myocardial or vascular injury, none of them which can be fully represented by any single conventional risk factor [10,11]. This interpretation aligns with evidence that CAV is initiated early after transplantation, with later clinical manifestations representing the culmination of sustained endothelial injury and immune-mediated remodeling [15,16]. Studies have previously established that early inflammatory markers, including C-reactive protein measured in the first post-transplant biopsy, have shown to independently predict CAV at 5 and 10 years [16]. Remarkably, patients who developed the composite endpoint had an increased Δ*Age* compared with event-free patients (−1.8 vs. −8.0 years), suggesting that an older electrocardiographic profile at discharge may supports this hypothesis that the pathophysiologic trajectory toward vasculopathy may be established at or before hospital discharge.

The pilot study of AI-ECG as a predictive model for CAV is novel, as the analysis was conducted in a small cohort (*n* = 50) with complete-case modeling; it can raise the possibility of overfitting and optimism bias. Therefore, bootstrap-based optimism correction is recommended as a minimum standard for internal validation. Our results with bootstrap-based internal validation across 1000 resamples demonstrated negligible optimism, with an optimism-corrected C-index identical to the apparent estimate (0.692), suggesting that the observed discrimination is unlikely to reflect spurious model performance in this cohort [17,18].

These findings carry potential clinical relevance, as a non-invasive biomarker capable of early CAV risk stratification which could meaningfully alter post-transplant management. While clinical application requires prospective validation in larger, multicenter cohorts, these pilot findings represent an early but promising step toward establishing discharge AI-ECG-derived age gap as a prognostic tool in a population for whom the need for better and earlier markers of allograft injury remains unmet.

### 4.1. Limitations

There are several limitations to this study. Firstly, due to the novelty of AI-ECG application, there was a small sample size (*n* = 50, with 16 events) posing a risk for generalizability and type 1 error [18,19,20]. Our population is unique due to the focus on patients bridged with Impella 5.5; however, given the variable nature of donor–recipient matching, and our center performance in line with the SRTR nationally expected parameters, our study may not be generalizable for centers that do not use the Impella 5.5 as a bridge to transplant strategy. The overlap in ΔAge distributions and moderate discrimination limit its utility for individual clinical decision-making but highlight the multi-modality assessment in prognostication for a population that has limited to no non-invasive markers currently. Lack of IVUS may be considered a limitation however, as it is not always performed routinely in all transplant centers. Additionally, genetic and lifestyle factors known to influence biological aging, including physical activity, diet, socioeconomic status, and inherited predisposition to accelerated vascular or cardiac aging, were not captured in this dataset and still remain unknown in their role of CAV after transplant while on immunotherapy. Pre-transplant effect of perioperative parameters on the potential influence on AI-ECG-derived ΔAge or CAV risk was not assessed and their impact after heart transplant remains unknown.

### 4.2. Future Directions

Future direction should aim to externally validate the findings in a larger, multicenter cohort with diverse patient populations. We can achieve higher prognostication value and mechanistic insights by adding donor variables, rejection burden, and time-to-event analyses with a Cox proportional hazards modeling. A multimodal approach could subsequently integrate AI-ECG features with donor characteristics, rejection burden, clinical risk factors, laboratory biomarkers, and cardiac imaging parameters to develop a better risk model for CAV. Prospective studies with external validation will be necessary to determine whether such multimodal models provide incremental discrimination and clinically meaningful improvement over established CAV surveillance strategies. The development of composite scores, following prospective studies to incorporate AI-ECG-derived ΔAge alongside molecular and imaging biomarkers, represents a promising avenue for precision medicine in transplant cardiology. We could see a future where risk stratification in the setting of AI ECG and multi-factor approach may limit the need for invasive testing in higher-risk populations.

## 5. Conclusions

AI-ECG-derived heart age gap at discharge is a novel and clinically meaningful predictor of cardiac allograft vasculopathy (CAV). Our study suggests that the biological trajectory toward CAV is established as early as the time of discharge, which can be identified non-invasively using standard ECG data. This represents a meaningful step toward precision risk stratification in transplant cardiology.

## Figures and Tables

**Table 1:** Baseline characteristics of the study cohort stratified by composite event status.

Variable	Event (*n* = 16)	No Event (*n* = 34)	Overall (*n* = 50)	SMD	*p*-Value
Recipient Characteristics					
Age, years	60 ± 11	59 ± 13	59 ± 13	0.13	0.618
Male sex, n (%)	14 (88)	28 (82)	42 (84)	0.16	0.693
Race/ethnicity, n (%)					
White	9 (56)	20 (59)	29 (58)	—	—
African American	6 (38)	12 (35)	18 (36)	—	—
Other	1 (6)	2 (6)	3 (6)	—	—
Ischemic cardiomyopathy, n (%)	7 (44)	12 (35)	19 (38)	0.18	0.548
Type 2 diabetes mellitus, n (%)	9 (56)	14 (41)	23 (46)	0.30	0.368
Hypertension, n (%)	11 (69)	22 (65)	33 (66)	0.09	1.000
Ex-smoker, n (%)	7 (44)	15 (44)	22 (44)	0.00	1.000
Admission LVEF, %	18 (14–23)	18 (13–22)	18 (14–22)	−0.04	0.938
Impella 5.5 support duration, days	22 (10–41)	21 (11–40)	22 (10–40)	0.02	0.963
Donor Characteristics					
Donor age, years	36 ± 7	32 ± 9	33 ± 8	0.54	0.061
AI-ECG Biomarker					
Discharge ΔAge, years	−1.8 ± 14.0	−8.0 ± 13.9	−6.1 ± 14.2	0.45	0.091
Outcomes					
Composite endpoint (CAV or death ≤3 yr), n (%)	16 (100)	0 (0)	16 (32)	—	—
CAV (ISHLT Grade 1–3), n (%)	12 (75)	0 (0)	12 (24)	—	—
All-cause mortality ≤3 yr, n (%)	4 (25)	0 (0)	4 (8)	—	—

SMD = standardized mean difference; LVEF = left ventricular ejection fraction; CAV = cardiac allograft vasculopathy; ΔAge = AI-ECG predicted age minus chronological age at hospital discharge.

**Table 2:** Cox proportional hazard regression: discharge ΔAge and clinical outcome.

Model/Variable	HR	95% CI	*p* -Value	Notes
Primary Endpoint: Composite (CAV or All-cause Mortality ≤3 Years)				
Univariable Cox regression				
Discharge ΔAge per 1 SD (14.2 years)	1.81	1.16–2.83	0.009	Continuous; *n* = 16 events
Parsimonious adjusted Cox (ridge-penalized, λ = 0.10)				
Discharge ΔAge per 1 SD	1.47	0.87–2.47	0.147	Adjusted: recipient age + donor age
Secondary Endpoint: CAV or Mortality				
Univariable Cox regression (CAV Alone)				
Discharge ΔAge per 1 SD	2.19	1.31–3.66	0.003	*n* = 12 CAV events; exploratory
Mortality Alone (Descriptive Only)				
All-cause mortality ≤3 yr	—	—	—	*n* = 4 events; insufficient for regression

HR = hazard ratio; CI = confidence interval; ΔAge = AI-ECG predicted age minus chronological age at discharge; SD = standard deviation.

**Table 3:** Model performance metrics and internal validation.

Performance Metric	Estimate	Notes/Interpretation
Discrimination		
Apparent C-index (Harrell)	0.692	Univariable; discharge ΔAge vs. composite
Optimism-corrected C-index	0.692	1000 bootstrap resamples; optimism = 0.000
3-Year AUC	0.716	95% CI 0.550–0.857 (bootstrap 1000 resamples)
Exploratory Youden threshold	ΔAge = −3.0 yr	Hypothesis-generating only; no validated cutoff
Proportional Hazard Assessment		
Schoenfeld global test *p*-value	0.657	No violation of PH assumption detected
Cohort Summary		
Sample size	*n* = 50	Single-center pilot cohort, 2020–2023
Composite events	16 (32%)	CAV or all-cause mortality within 3 years
CAV events	12 (24%)	ISHLT Grade 1–3
All-cause mortality ≤3 yr	4 (8%)	Insufficient for independent regression
Median follow-up	3.0 years	Reverse Kaplan–Meier estimator
Events per variable (EPV)	16/1	Supports univariable model only

AUC = area under the receiver operating characteristic curve; EPV = events per variable; PH = proportional hazards.

## Data Availability

The ECG and clinical data presented in this study are available on request from the corresponding author due to patient privacy and institutional data-use restrictions.

## Abbreviations

The following abbreviations are used in this manuscript:

AI-ECG	Artificial Intelligence Electrocardiography
AU	Area Under the Curve
CAV	Cardiac Allograft Vasculopathy
CI	Confidence Interval
ECG	Electrocardiogram
EPVs	Events Per Variable
HR	Hazard Ratio
IQR	Interquartile Range
IRB	Institutional Review Board
ISHLT	International Society for Heart and Lung Transplantation
LVEF	Left Ventricular Ejection Fraction
PHs	Proportional Hazards
Q-Q plot	Quantile-Quantile Plot
ROC	Receiver Operating Characteristic
SD	Standard Deviation
SMD	Standardized Mean Difference
ΔAge (Delta Age)	AI-ECG Predicted Age minus Chronological Age

## References

[1] Virani S.S., Newby L.K., Arnold S.V., Bittner V., Brewer L.C., Demeter S.H., Dixon D.L., Fearon W.F., Hess B., Johnson H.M. (2023). 2023 AHA/ACC/ACCP/ASPC/NLA/PCNA Guideline for the Management of Patients With Chronic Coronary Disease: A Report of the American Heart Association/American College of Cardiology Joint Committee on Clinical Practice Guidelines. Circulation.

[2] Clerkin K.J., Topkara V.K., Farr M.A., Jain R., Colombo P.C., Restaino S., Sayer G., Castillo M., Lam E.Y., Chernovolenko M. (2022). Noninvasive Physiologic Assessment of Cardiac Allograft Vasculopathy Is Prognostic for Post-Transplant Events. J. Am. Coll. Cardiol..

[3] Chih S., Chong A.Y., Erthal F., DeKemp R.A., Davies R.A., Stadnick E., So D.Y., Overgaard C., Wells G., Mielniczuk L.M. (2018). PET Assessment of Epicardial Intimal Disease and Microvascular Dysfunction in Cardiac Allograft Vasculopathy. J. Am. Coll. Cardiol..

[4] Belmonte M., Paolisso P., Viscusi M.M., Beles M., Bergamaschi L., Sansonetti A., Ohashi H., Seki R., Gallinoro E., Esposito G. (2025). Comprehensive Non-invasive Versus Invasive Approach to Evaluate Cardiac Allograft Vasculopathy in Heart Transplantation: The CCTA-HTx Study. Circ. Cardiovasc. Imaging.

[5] Ortega-Legaspi J.M., Bravo P.E. (2022). Diagnosis and management of cardiac allograft vasculopathy. Heart.

[6] Madamanchi C., Konerman M.C., Murthy V.L. (2021). Imaging Coronary Allograft Vasculopathy with Cardiac PET and Cardiac MRI. Curr. Cardiol. Rep..

[7] Adedinsewo D., Hardway H.D., Morales-Lara A.C., Wieczorek M.A., Johnson P.W., Douglass E.J., Dangott B.J., Nakhleh R.E., Narula T., Patel P.C. (2023). Non-invasive detection of cardiac allograft rejection among heart transplant recipients using an electrocardiogram based deep learning model. Eur. Heart J. Digit. Health.

[8] Loupy A., Sablik M., Khush K., Reese P.P. (2025). Advancing patient monitoring, diagnostics, and treatment strategies for transplant precision medicine. Lancet.

[9] Holzhauser L., DeFilippis E.M., Nikolova A., Byku M., Contreras J.P., De Marco T., Hall S., Khush K.K., Vest A.R. (2023). The End of Endomyocardial Biopsy?. JACC Heart Fail..

[10] Lopez-Jimenez F., Kapa S., Friedman P.A., LeBrasseur N.K., Klavetter E., Mangold K.E., Attia Z.I. (2024). Assessing Biological Age. JACC Clin. Electrophysiol..

[11] Mossavarali S., Vaezi A., Gholami Z., Molaei A., Yekaninejad M.S., Asselbergs F.W., Shafiee A. (2025). Determinants of artificial intelligence electrocardiogram-derived age and its association with cardiovascular events and mortality: A systematic review and meta-analysis. npj Digit Med..

[12] Attia Z.I., Friedman P.A., Noseworthy P.A., Lopez-Jimenez F., Ladewig D.J., Satam G., Pellikka P.A., Munger T.M., Asirvatham S.J., Scott C.G. (2019). Age and Sex Estimation Using Artificial Intelligence From Standard 12-Lead ECGs. Circ. Arrhythmia Electrophysiol..

[13] Peled Y., Ducharme A., Kittleson M., Bansal N., Stehlik J., Amdani S., Saeed D., Cheng R., Clarke B., Dobbels F. (2024). International Society for Heart and Lung Transplantation Guidelines for the Evaluation and Care of Cardiac Transplant Candidates—2024. J. Heart Lung Transplant..

[14] Hashim H.T., Ramadhan M.A., Ahmad S., Shah J., Varney J., Motawea K.R., Kandil O.A. (2022). The role of the electrocardiogram in the recognition of cardiac transplant rejection: A systematic review and meta-analysis. Clin. Cardiol..

[15] David P., Roquero P., Coutance G. (2026). New Insights Into Pathogenesis, Diagnosis, and Management of Cardiac Allograft Vasculopathy. Can. J. Cardiol..

[16] Labarrere C.A., Woods J.R., Hardin J.W., Jaeger B.R., Zembala M., Deng M.C., Kassab G.S. (2014). Early Inflammatory Markers Are Independent Predictors of Cardiac Allograft Vasculopathy in Heart-Transplant Recipients. PLoS ONE.

[17] Noma H., Shinozaki T., Iba K., Teramukai S., Furukawa T.A. (2021). Confidence intervals of prediction accuracy measures for multivariable prediction models based on the bootstrap-based optimism correction methods. Stat. Med..

[18] Iba K., Shinozaki T., Maruo K., Noma H. (2021). Re-evaluation of the comparative effectiveness of bootstrap-based optimism correction methods in the development of multivariable clinical prediction models. BMC Med. Res. Methodol..

[19] Smith G.C.S., Seaman S.R., Wood A.M., Royston P., White I.R. (2014). Correcting for Optimistic Prediction in Small Data Sets. Am. J. Epidemiol..

[20] Poldrack R.A., Huckins G., Varoquaux G. (2020). Establishment of Best Practices for Evidence for Prediction: A Review. JAMA Psychiatry.

